# Supplementary material for: Conversion of Glycosylated Platycoside E to Deapiose-Xylosylated Platycodin D by Cytolase PCL5
Source: Int J Mol Sci. 2020 Feb 11;21(4):1207. doi: 10.3390/ijms21041207 (PMC7072768; doi:10.3390/ijms21041207)
Supplement: Supplementary file 1 [file ijms-21-01207-s001.pdf]

Conversion of Glycosylated Platycoside E to Deapiose-Xylosylated Platycodin D by Cytolase PCL5

Kyung-Chul Shin [1, †], Dae Wook Kim[2,†], Hyun Sim Woo[2], Deok-Kun Oh[1,3], Yeong-Su Kim[2,*]

[1] Research Institute of Bioactive-Metabolome Network, Konkuk University, Seoul 05029, Korea; hidex2@naver.com (K.-C.S.); deokkun@konkuk.ac.kr (D.-K.O.)
[2] Forest Plant Industry Department, Baekdudaegan National Arboretum, Bonghwa 36209, Korea; dwking@bdna.or.kr (D.W.K.); whs0428@bdna.or.kr (H.S.W.)
[3] Department of Bioscience and Biotechnology, Konkuk University, Seoul 05029, Korea; deokkun@konkuk.ac.kr (D.-K.O.)
* Correspondence: yskim@bdna.or.kr; Tel.: +82-54-679-2740; Fax: +82-54-679-0636
† These authors contributed equally to this work.

Supplementary Materials

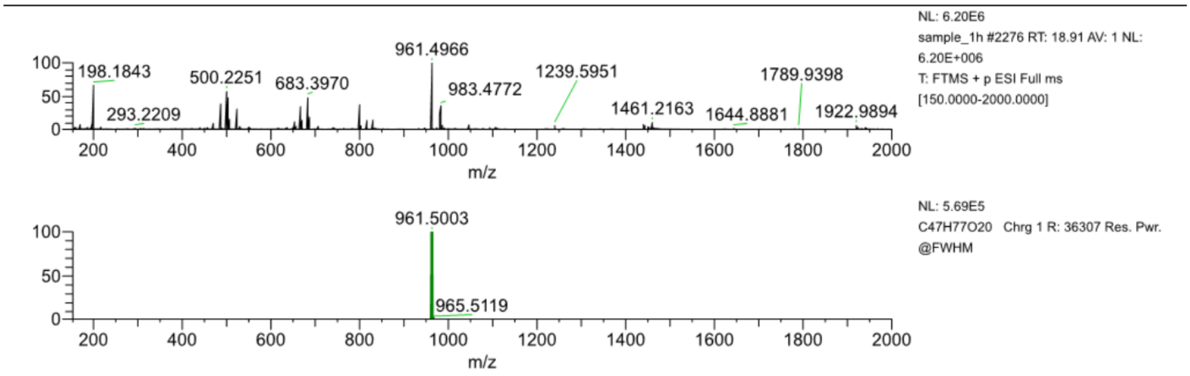

Display	File Name	Detector...	Filter	Trace Ty...	Ranges	Smoothi...	Delay Ti...	Reference	Plot Ope...	Trace Ty...	Range2	Comment
True	G:\1907...	MS		TIC		None	0	False				

Index	m/z	Formula	Score	RDB	Delta ppm	OriginalFormula	Theo. Mass
1	961.4966	$C_{47}H_{77}O_{20}$	88.2892529843...	9.5	-3.77	C47H77O20	961.50027
2		$C_{54}H_{73}O_{15}$	78.8906328156...	18.5	2.34	C54H73O15	961.49440
3		$C_{40}H_{81}O_{25}$	30.7046751588...	0.5	-9.88	C40H81O25	961.50614
4		$C_{61}H_{69}O_{10}$	16.5483883676...	27.5	8.44	C61H69O10	961.48852
5		$C_{65}H_{69}O_{7}$	0	31.5	-7.42	C65H69O7	961.50378
6		$C_{72}H_{65}O_{2}$	0	40.5	-1.32	C72H65O2	961.49791

Figure S1. LCMS and HRMS analysis of deapi-dexyl-platycodin D (**1′**).

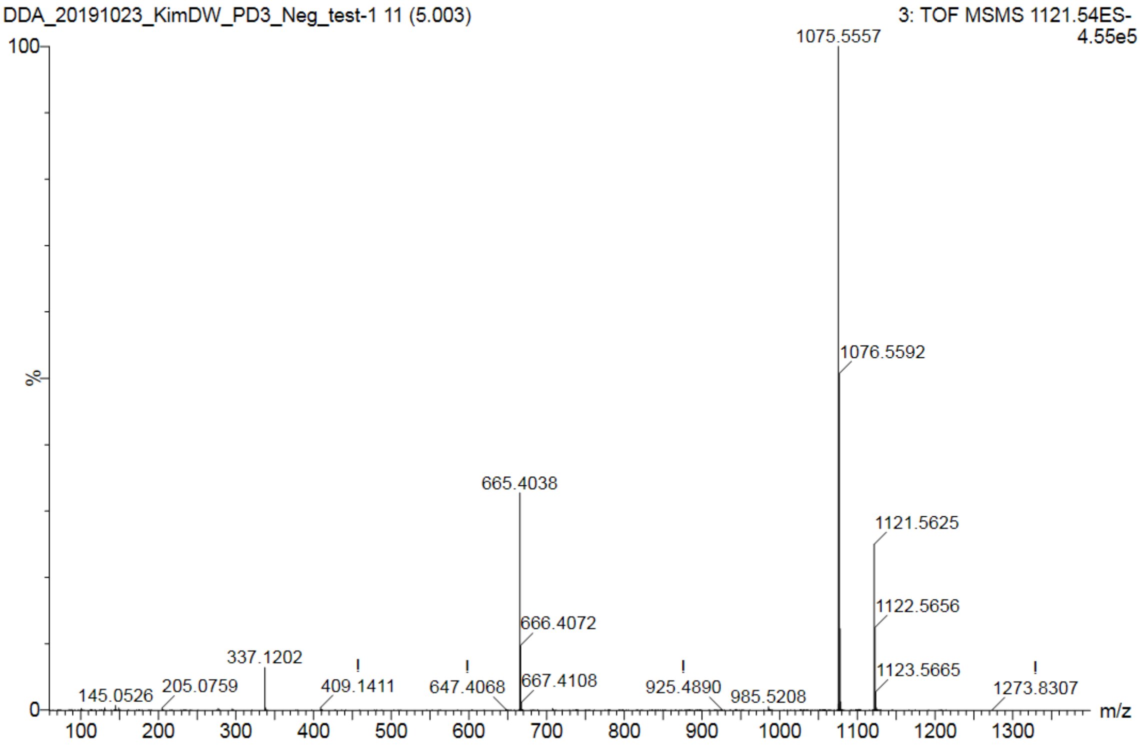

Elemental Composition Report

Tolerance = 5.0 PPM / DBE: min = -1.5, max = 20.0
Element prediction: Off
Number of isotope peaks used for i-FIT = 5

Monoisotopic Mass, Even Electron Ions
485 formula(e) evaluated with 2 results within limits (up to 20 closest results for each mass)
Elements Used:
C: 0-100 H: 0-200 O: 0-50

Minimum:	60.00				-1.5				
Maximum:	100.00		5.0	5.0	20.0				
Mass	RA	Calc. Mass	mDa	PPM	DBE	i-FIT	Norm	Conf(%)	Formula
1121.5436	100.00	1121.5439	-0.3	-0.3	2.5	212.6	0.300	74.06	C46 H89 O30
		1121.5380	5.6	5.0	11.5	213.6	1.349	25.94	C53 H85 O25

Figure S2. LCMS and HRMS analysis of deapi-patycodin D (**2′**).

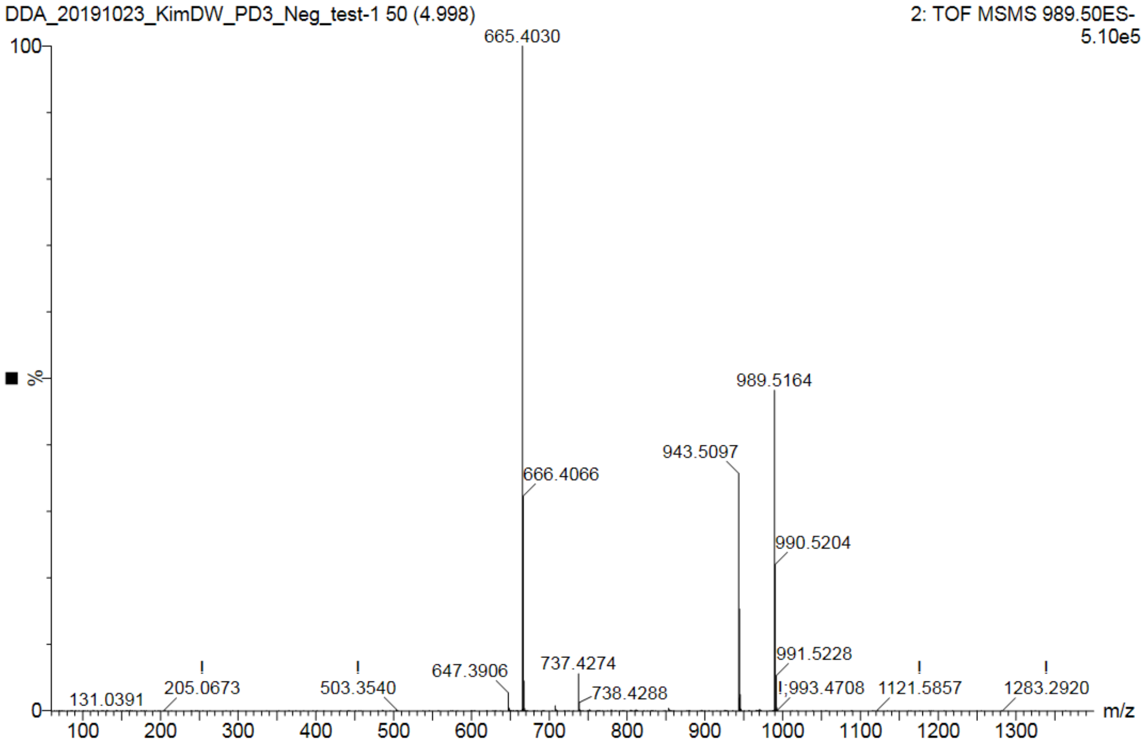

Elemental Composition Report

Tolerance = 5.0 PPM / DBE: min = -1.5, max = 20.0
Element prediction: Off
Number of isotope peaks used for i-FIT = 5

Monoisotopic Mass, Even Electron Ions
406 formula(e) evaluated with 2 results within limits (up to 20 closest results for each mass)
Elements Used:
C: 0-100 H: 0-200 O: 0-50

Minimum:	60.00				-1.5				
Maximum:	100.00		5.0	5.0	20.0				
Mass	RA	Calc. Mass	mDa	PPM	DBE	i-FIT	Norm	Conf(%)	Formula
989.4985	100.00	989.4957	2.8	2.8	10.5	221.4	0.853	42.60	C48 H77 O21
		989.5016	-3.1	-3.1	1.5	221.1	0.555	57.40	C41 H81 O26

Figure S3. LCMS and HRMS analysis of deapi-dexyl-platycodin D (**3′**)
